# Regulation of *HK2* expression through alterations in CpG methylation of the *HK2* promoter during progression of hepatocellular carcinoma

**DOI:** 10.18632/oncotarget.9723

**Published:** 2016-05-30

**Authors:** Hyun Gyu Lee, Hyemi Kim, Taekwon Son, Youngtae Jeong, Seung Up Kim, Seung Myung Dong, Young Nyun Park, Jong Doo Lee, Jae Myun Lee, Jeon Han Park

**Affiliations:** ^1^ Department of Microbiology and Immunology, Institute for Immunology and Immunological Diseases, Yonsei University College of Medicine, Seoul, Republic of Korea; ^2^ Brain Korea 21 PLUS Project for Medical Sciences, Yonsei University College of Medicine, Seoul, Republic of Korea; ^3^ Research Institute of Pharmaceutical Sciences, College of Pharmacy, Seoul National University, Seoul, Republic of Korea; ^4^ Stanford Cancer Institute and Institute for Stem Cell Biology and Regenerative Medicine, Stanford University School of Medicine, Stanford, California, USA; ^5^ Department of Internal Medicine, Institute of Gastroenterology, Yonsei University College of Medicine, Seoul, Republic of Korea; ^6^ Research Institute, National Cancer Center, Goyang, Gyeonggi-do, Republic of Korea; ^7^ Department of Pathology, Yonsei University College of Medicine, Seoul, Republic of Korea; ^8^ Department of Nuclear Medicine, International St. Mary's Hospital, Catholic Kwandong University College of Medicine, Incheon, Republic of Korea

**Keywords:** hexokinase 2, HumanMethylation450 BeadChip, HK2-CIMP, hypoxia response element, HIF-1α

## Abstract

Hexokinase 2 (HK2) is a rate-determining enzyme in aerobic glycolysis, a process upregulated in tumor cells. *HK2* expression is controlled by various transcription factors and epigenetic alterations and is heterogeneous in hepatocellular carcinomas (HCCs), though the cause of this heterogeneity is not known. DNA methylation in the *HK2* promoter CpG island (*HK2*-CGI) and its surrounding regions (shore and shelf) has not previously been evaluated, but may provide clues about the regulation of HK2 expression. Here, we compared *HK2* promoter methylation in HCCs and adjacent non-cancerous liver tissues using a HumanMethylation450 BeadChip array. We found that, while the *HK2*-CGI N-shore was hypomethylated, thereby enhancing *HK2* expression, the *HK2*-CGI was itself hypermethylated in some HCCs. This hypermethylation suppressed *HK2* expression by inhibiting interactions between HIF-1α and a hypoxia response element (HRE) located at −234/−230. HCCs that were HK2^negative^ and had distinct promoter CGI methylation were denoted as having a *HK2*-CGI methylation phenotype (*HK2*-CIMP), which was associated with poor clinical outcome. These findings indicate that *HK2*-CGI N-shore hypomethylation and *HK2*-CGI hypermethylation affect *HK2* expression by influencing the interaction between HIF 1α and HRE. *HK2*-CGI hypermethylation induces *HK2*-CIMP and could represent a prognostic biomarker for HCC.

## INTRODUCTION

Hexokinase 2 (HK2) is a rate-determining enzyme in aerobic glycolysis. *HK2* is rarely expressed in normal tissues, except skeletal and cardiac muscle and adipose tissues [1]; however, it is frequently upregulated in tumor cells, leading to a phenomenon known as the Warburg Effect. In spite of frequent *HK2* expression in various cancers [2], hepatocellular carcinomas (HCCs) exhibit heterogeneous expression of *HK2* [3–5], which contributes to heterogeneous ^18^F-2-fluoro-2-deoxy-D-glucose (^18^F-FDG) uptake in positron emission tomography (PET) scan [6, 7] and therefore reduces the clinical usefulness of ^18^F-FDG PET in HCCs [8, 9]. However, the regulation of *HK2* expression in HCCs still remains elusive [10]. Defining the mechanisms underlying *HK2* expression could give a clue about the heterogeneous expression of *HK2* in HCCs.

Various transcription factors and microRNAs are involved in the regulation of *HK2* expression during cancer initiation and progression [11, 12]. Hypoxia-inducible factor-1α (HIF-1α) induces aggressive tumor phenotypes by regulating more than 60 target genes, including *HK2* [13]. Recently, HIF-1α was implicated in the indirect regulation of *HK2* expression via the suppression of miR-199a-5p [14]. Despite these findings, little is known about how HIF-1α directly regulates *HK2* expression [15, 16].

CpG methylation, an epigenetic modification characterized by a substitution of cytosine-C5 with a methyl-cytosine in CpG dinucleotides, regulates gene transcription [17]. A recent report has suggested that gene expression is regulated by the alteration of CpG methylation in the promoter CpG island (CGI) shore (up to 2 kb upstream from CGI), rather than in the promoter CGI itself [18, 19]. Most CGIs in normal tissues remain largely unmethylated [20, 21], but unknown factors during cancer initiation and progression cause *de novo* methylation [22], perhaps by crosstalk between DNA methyltransferases (DNMTs) and histone methyltransferases (HMTs) [22, 23]. *HK2* expression is regulated by CpG methylation [24, 25], but it is unclear whether these alterations occur in the *HK2* promoter CGI (referred to as *HK2*-CGI) or its shore. This knowledge may help to elucidate the precise molecular mechanism of *HK2* expression.

In this study, to determine why *HK2* is heterogeneously expressed in HCCs, we compared *HK2* promoter methylation in HCCs and adjacent non-cancerous liver tissues (Adj-NCLs) using the HumanMethylation450 BeadChip (HM450) array. We evaluated how those methylation changes were influenced by DNMTs and HMTs using HCC cell lines with differential *HK2* expression. We also identified a key regulatory region for expression of *HK2* by dissecting the differentially methylated regions in the *HK2* promoter and evaluated how these methylation changes influence *HK2* expression. Finally, we demonstrated that HCCs with specific and significant methylation changes could be regarded as a phenotypic HCCs subgroup.

## RESULTS

### *HK2*-CGI hypermethylation in HK2^negative^ HCCs

To identify the mechanism underlying *HK2* expression, we assessed HCCs and Adj-NCLs for differences in CpG methylation in the *HK2* promoter. We initially performed bisulfite sequencing and pyrosequencing. However, the dense CpGs in the *HK2*-CGI region made it difficult to analyze the methylation status of that region. Thus, we conducted a comparative methylation analysis using the HM450 array on 24 HCCs and 18 Adj-NCLs. This analysis revealed global hypomethylation (Supplementary Figure S1A) in the HCCs, which discriminated them from Adj-NCLs in an unsupervised hierarchical clustering analysis (14/24, 58.3%; Supplementary Figure S1B). The branch lengths were longer in HCCs than in Adj-NCLs, indicating that HCCs are more heterogeneously methylated (Supplementary Figure S1B).

**Figure 1:**
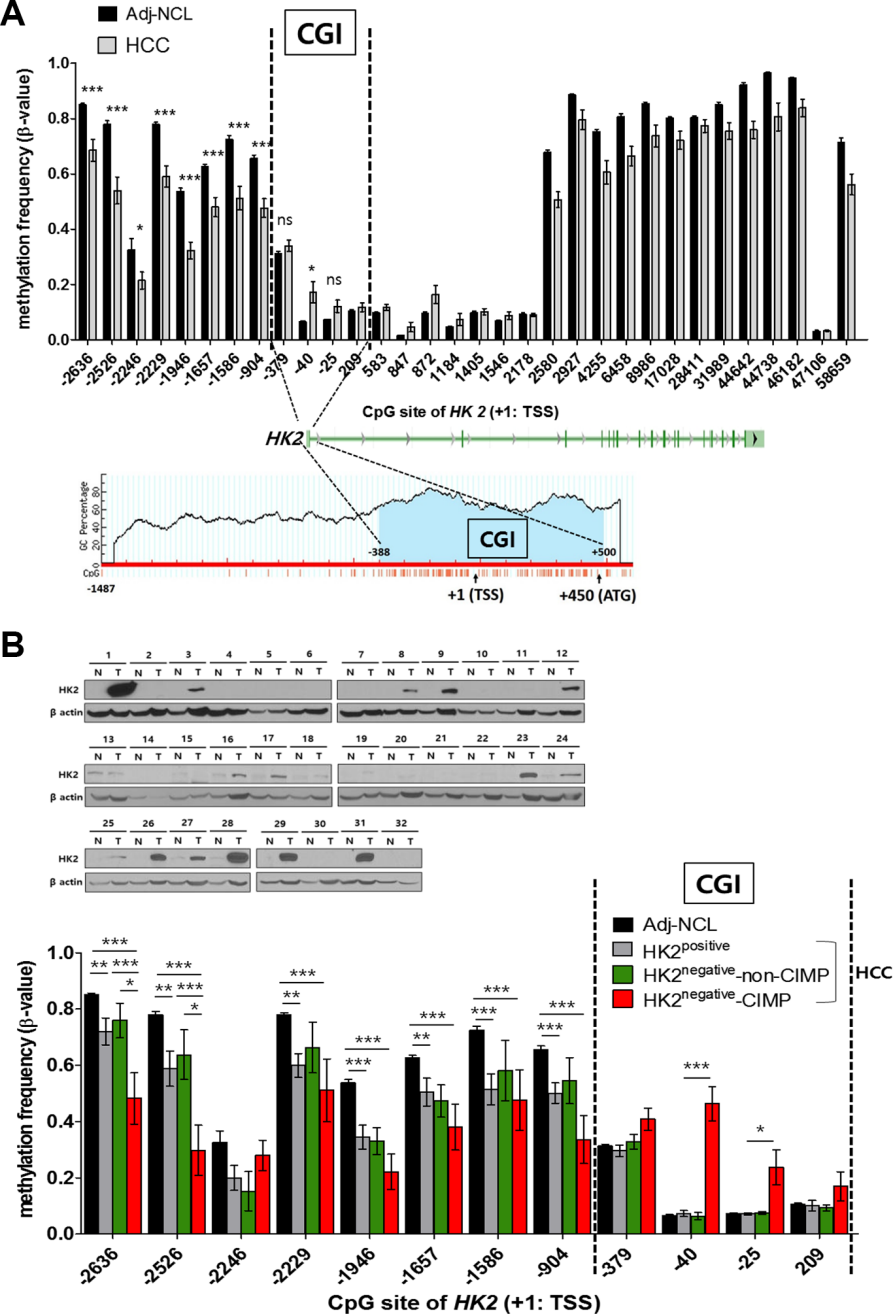
Two different alterations of CpG DNA methylation in the *HK2* promoter: hypomethylation at the *HK2*-CGI N-shore and hypermethylation at the *HK2*-CGI observed only in HK2^negative^*HK2*-CIMP HCCs (**A**) A comparison of *HK2* promoter methylation status between HCC and Adj-NCL tissues. The *HK2*-CGI located at the range of −388 to +500 bp from the transcriptional start site (TSS: +1) was predicted by Methprimer (http://www.urogene.org/cgi-bin/methprimer/methprimer.cgi, lower panel). (**B**) The methylation status of the *HK2* promoter according to HK2 expression in HCC tissues. HK2^positive^ or HK2^negative^ was defined by immunoblot. The β-values of CpGs in the *HK2* promoter were plotted. Two vertically dashed line indicted the borders of the *HK2*-CGI. Values represent the mean ± SEM. *P < 0.05, **P < 0.01, ***P < 0.001, ns, not significant.

We also observed an altered methylation profile in and around the *HK2*-CGI (Figure 1A). In Adj-NCLs, most regions of the *HK2* promoter were densely methylated, while HCC tissues were hypomethylated, particularly in the *HK2*-CGI N-shore (Figure 1A upper panel). On the contrary, the *HK2*-CGI was sparsely methylated in Adj-NCLs, while the −40 CpG site in the *HK2*-CGI was hypermethylated in HCCs (*P* = 0.0372, Figure 1A upper panel).

Because altered methylation in the *HK2*-CGI and its N-shore should correlate with the regulation of *HK2* expression, we investigated the relationship of methylation status of *HK2* promoter with HK2 protein expression (Figure 1B upper panel). Interestingly, we observed −40 CpG hypermethylation in some HK2^negative^ HCCs; the −25 CpG was hypermethylated in these cells as well (*P* = 0.0324, Figure 1B lower panel and Supplementary Figure S2A). This *HK2*-CGI hypermethylation profile was not found in any other HCCs tested, thus we grouped this subset of HCCs by their CGI methylation phenotype, naming them *HK2*-CIMP (Figure 1B and Supplementary Figure S2B). The *HK2*-CGI N-shore was hypomethylated in the HK2^positive^ and HK2^negative^-non-CIMP HCCs, but there were no significant differences between these two groups (Figure 1B lower panel). In this same region, *HK2*-CIMP HCCs showed the most robust hypomethylation in the N-shore (Figure 1B lower panel). Taken together, these data indicate that most regions of the *HK2* promoter are relatively hypomethylated in HCCs compared to Adj- NCL, which is consistent with previous reports [26, 27]. However, the *HK2*-CGI was hypermethylated in a subset of cells, the *HK2*-CIMP HCCs, which do not express HK2 protein.

### The correlation of HK2 suppression and *HK2*-CGI hypermethylation

**Figure 2:**
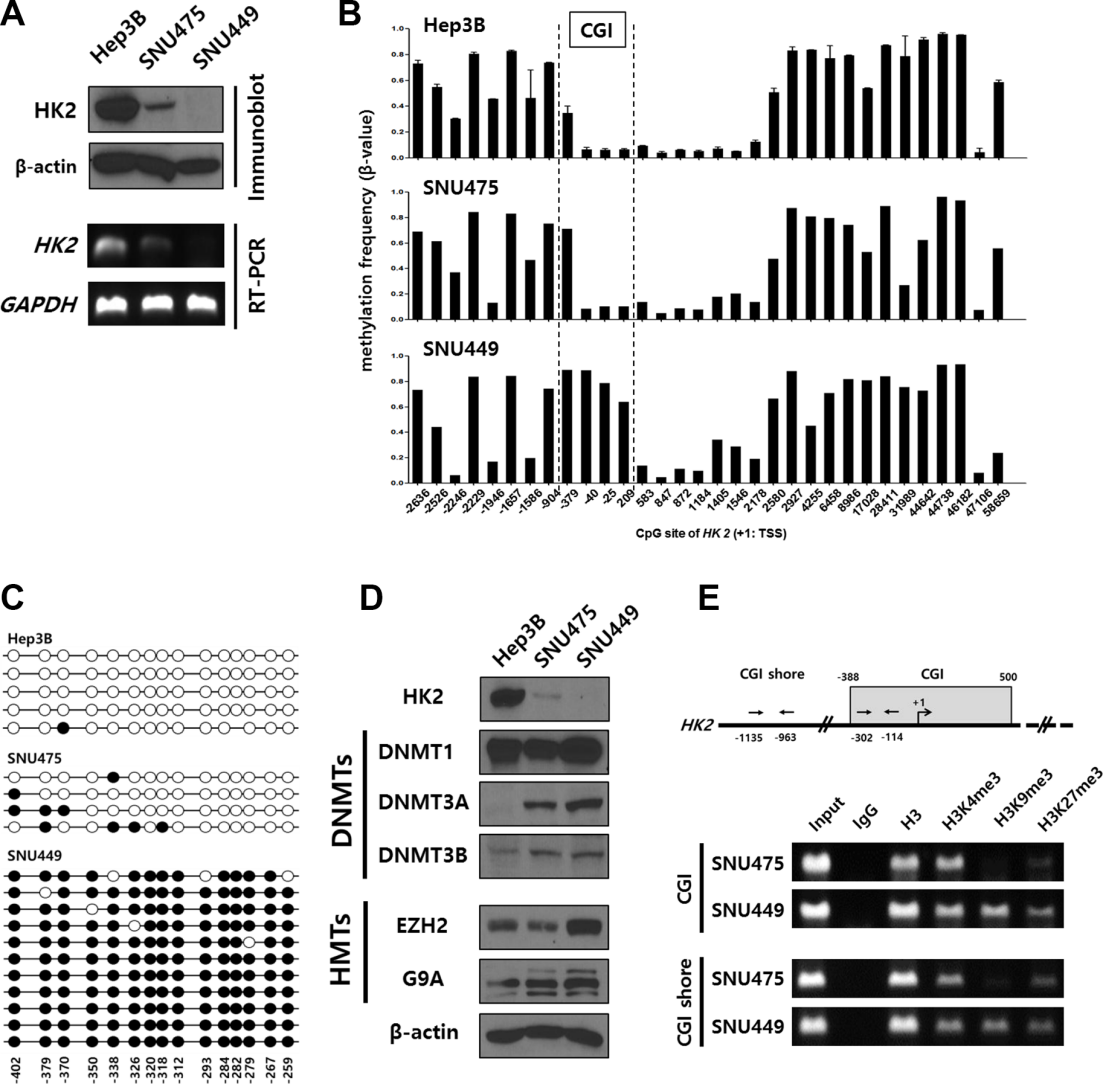
Hypermethylation of the *HK2*-CGI caused by the crosstalk of DNMTs and HMTs suppresses *HK2* expression in HCC cell lines (**A**) HK2 protein expression in HCC cell lines (Hep3B, SNU475, and SNU449 cells) was evaluated by immunoblot and RT-PCR. (**B**) Methylation status of *HK2* promoter among HCC cell lines. The *HK2*-CGI is indicated by dashed lines. (**C**) Bisulfite sequencing of the *HK2*-CGI in Hep3B, SNU475, and SNU449 cells. Each circle indicates an individual CpG site. An individual row represents a single clone. The open and closed circles denote unmethylated and methylated CpG sites, respectively. (**D**) DNMTs (DNMT1, DNMT3A, and DNMT3B) and HMTs (EZH2 and G9A) were evaluated in HCC cell lines by immunoblot. (**E**) The chromatin status of indicated cell lines was evaluated by H3K4me3-, H3K9me3-, and H3K27me3-ChIP assay.

To determine whether *HK2*-CGI hypermethylation was responsible for HK2 suppression in *HK2*-CIMP HCCs, we examined HK2 protein expression in the HCC cell lines: Hep3B, SNU449, and SNU475. Hep3B cells expressed HK2 more abundantly than did SNU475 cells, while SNU449 cells did not express detectable levels of HK2 (Figure 2A upper panel). These results were consistent with RT-PCR results from the same cell lines (Figure 2A lower panel). Next, we assessed the methylation status of *HK2* in these cell lines using the HM450 array. Hep3B and SNU475 cells had a hypomethylated N-shore of the *HK2*-CGI, consistent with results from the HK2^positive^ HCC tissues tested. The *HK2*-CGI of those cells was not methylated, while that of SNU449 cells was densely methylated (Figure 2B). We conducted bisulfite sequencing to confirm the methylation status of the *HK2*-CGI. Consistent with the HM450 array data, the *HK2*-CGI of SNU449 cells was densely methylated, while in SNU475 and HepB3 cells it was not (Figure 2C).

To verify the factors that contribute to hypermethylation in the *HK2*-CGI, we measured the protein expression of DNA methyltransferases (DNMTs) and histone methyltransferases (HMTs) in each cell line using immunoblots. We observed slight differences in DNMT1 expression between the cell lines; however, DNMT3A and DNMT3B were more abundant in SNU449 cells than in Hep3B and SNU475 cells (Figure 2D upper panel). HMTs, such as Enhancer of Zeste 2 (EZH2) and Euchromatic histone-lysine N-methyltransferase 2 (EHMT2 or G9A), are associated with the recruitment of DNMT3s [22]. Expression of both of these proteins was increased in SNU449 cells (Figure 2D lower panel).

Furthermore, we evaluated histone lysine methylation status such as tri-methylation of lysine 4, lysine 9, and lysine 27 on histone 3 (H3K4me3, H3K9me3, and H3K27me3, respectively) to determine the status of chromatin modifications near the *HK2* gene. The histone lysine methylation status of SNU475 cells indicated active chromatin (H3K4me3 high, H3K9me3 low, and H3K27me3 low) in the *HK2*-CGI, while that of SNU449 cells represented a poised chromatin status (H3K4me3 high and H2K27me3 high) at the same region (Figure 2E). Moreover, the histone lysine methylation status of the *HK2*-CGI N-shore was similar to that of the HK2-CGI (Figure 2E). These data suggest that SNU449 cells are representative of *HK2*-CIMP HCCs with respect to HK2 expression and methylation status of the *HK2* promoter. The presence of H3K9me3 and H3K27me3 and the increased expression of DNMT3s, EZH2, and G9A suggest that *HK2*-CGI hypermethylation may result from crosstalk between DNMT3s and HMTs to alter *HK2*-CGI methylation and *HK2* expression.

### Identification of the −234/−230 HRE in the *HK2* promoter

**Figure 3:**
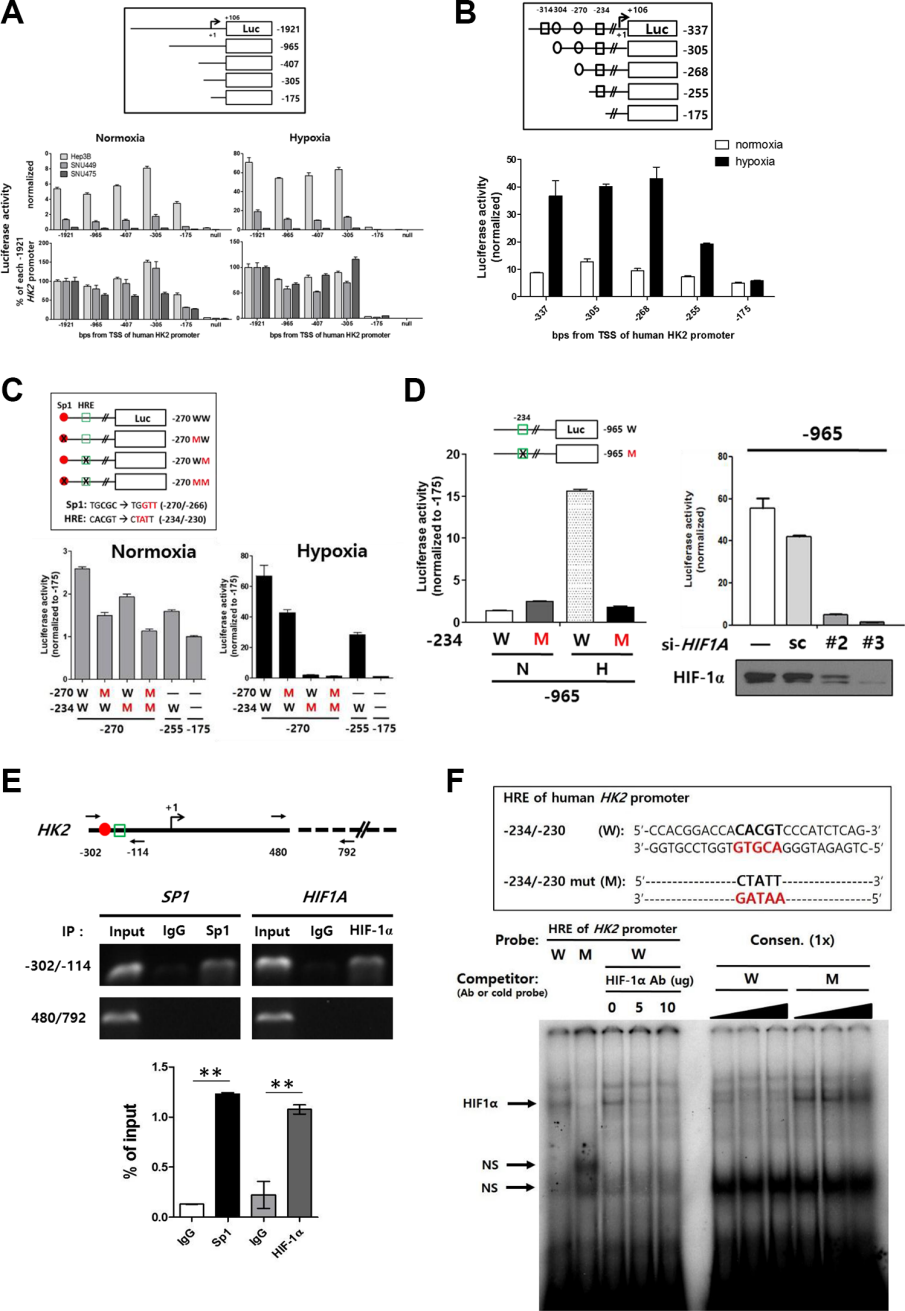
Identification of −234/−230 HRE, a key region in the *HK2* promoter regulated by methylation (**A**) The promoter activity of *HK2* promoter-deleted luciferase constructs was evaluated in HCC cell lines under normoxic and hypoxic conditions. Luciferase activity of all test constructs was normalized to that of the null construct. The relative luciferase activity was plotted as the percentage of the -1921 construct under normoxic or hypoxic conditions. (**B**) The promoter activity of *HK2* promoter-deleted luciferase constructs under normoxic or hypoxic conditions using Hep3B cells (rectangle; putative HRE, circle; putative Sp1-binding site). (**C**) The *HK2* promoter site-specific mutant luciferase constructs for putative HIF-1α and Sp1 binding sites were constructed as described in the upper panel. The promoter activity of each mutant construct under normoxic or hypoxic conditions is shown as the luciferase activity relative to the −175 construct (red closed circle; putative Sp1-binding site, green open rectangle; putative HRE). (**D**) The luciferase activity of the −965W and −965M under normoxic (N) or hypoxic (H) conditions. (**E**) The interaction between the −234/−230 HRE and HIF-1α was evaluated by ChIP assay. Sp1 was used as a positive control. The −302/−114 region and a non-relevant region (480/792) designed as shown in the upper panel were amplified by PCR. The specific interaction was plotted as the percentage of the input in the lower panel. (**F**) EMSA experiment involving the −234/−230 HRE and the mutant version (HREm) on the human *HK2* promoter. The oligonucleotides shown in the upper panel were labeled and incubated with nuclear extracts from Hep3B cells. NS, non-specific bands.

Although our observations suggested that *HK2* expression was largely regulated by epigenetic alterations, we sought to identify any cis-acting regions and corresponding trans-acting elements that also contribute to the regulation of *HK2* expression. A series of *HK2* promoter luciferase deletion constructs were generated to map the promoter regions that regulate *HK2* expression (Figure 3A upper panel). The luciferase activity of most constructs was increased in response to hypoxic stimuli. Interestingly, the −175 construct had decreased luciferase activity (Figure 3A lower panel), suggesting that the promoter region between −305 to −175 is an important regulatory region. Thus, additional luciferase constructs were generated using the Transcriptional Element Search System (http://www.cbil.upenn.edu/tess) (Figure 3B upper panel). When the luciferase activity of each construct was analyzed in Hep3B cells, activity was profoundly decreased in the −255 and −175 constructs under hypoxic conditions (Figure 3B lower panel). Further analysis suggested putative Sp1 and HIF-1α binding sites in−270 and −234 region.

We therefore performed luciferase assays with three kinds of −270/−234 site-specific mutants (MW, WM, and MM; Figure 3C upper panel) in Sp1 and/or HIF-1α binding sites. Relative decreases of luciferase activity among mutants were observed under normoxic conditions. Luciferase activity of the HRE mutant (WM) decreased to baseline under hypoxic conditions (Figure 3C lower panel). To determine whether the −234/−230 putative HRE acts as a key regulatory region for *HK2* expression, the −234/−230 HRE was mutated in the −965 construct (−965M). Luciferase activity of −965M was decreased to baseline under hypoxic conditions (Figure 3D left panel). Moreover, luciferase activity of −965W was decreased by silencing HIF-1α using RNA interference (Figure 3D right panel).

Next, we tested whether HIF-1α interacts with this HRE. We immunoprecipitated nuclear extracts of hypoxia-treated Hep3B cells with the anti-HIF-1α antibody (Ab) and observed a strong enrichment of the *HK2*-CGI region (−302/−114 PCR product) (Figure 3E). Finally, we confirmed a direct interaction between HIF-1α and −234/−230 HRE using electrophoretic mobility shift assays (EMSAs). The −234/−230 HRE consensus sequence probe showed a specific interaction with the nuclear extract, while its mutant probe did not (Figure 3F). Moreover, cold −234/−230 HRE probe inhibited the mobility shift of the −234/−230 HRE consensus sequence probe in a dose-dependent manner. The same inhibition was observed when anti-HIF-1α Ab was applied (Figure 3F). Taken together, these data show that HK2 expression is regulated by an interaction between HIF-1α and the −234/−230 HRE in the *HK2*-CGI.

### Induced hypomethylation of the HRE in the *HK2*-CGI allows HIF-1α binding and HK2 expression

**Figure 4:**
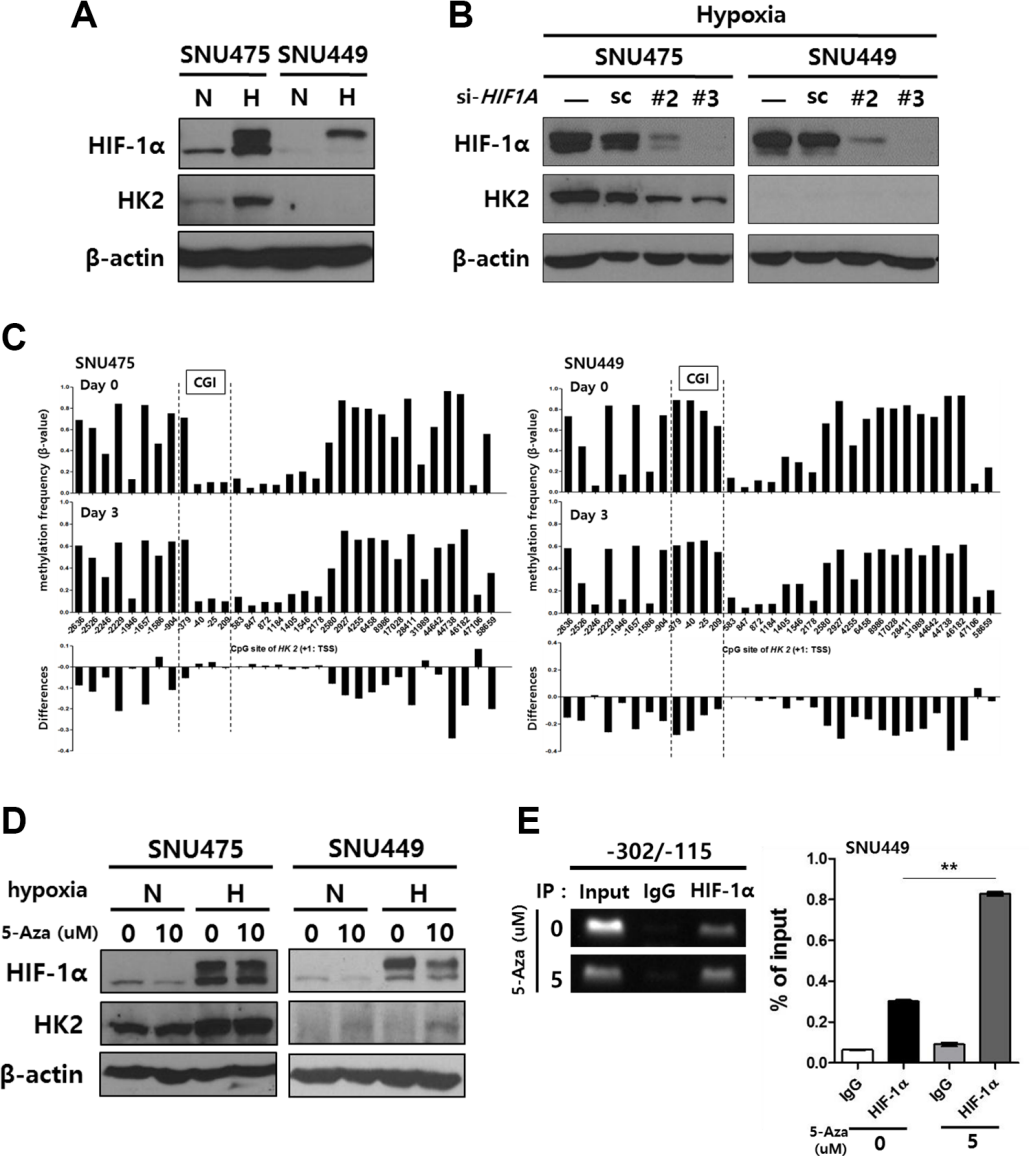
The induction of HK2 expression in HK2negative SNU449 cells by treatment with 5-Aza-CdR and hypoxia (**A**) Hypoxia-mediated HK2 expression. (**B**) The suppression of HK2 expression by HIF-1α silencing. (**C**) The methylation status of the *HK2* promoter CpGs plotted for the 5-Aza-CdR-treated SNU475 cells and SNU449 cells. The difference in methylation frequency between 5-Aza-CdR-treated cells and untreated cells is shown in each lower panel. (**D**) The induction of HK2 expression in SNU475 and SNU449 cells by treatment with 5-Aza-CdR for 2 d, followed by hypoxic stimuli for 1 d. In all experiments, the expression of HIF-1α and HK2 were evaluated by immunoblot. (**E**) The interaction between the −234/−230 HRE and HIF-1α following 5-Aza-CdR treatment was evaluated using a ChIP assay.

We next investigated the influence of *HK2*-CGI methylation on HK2 expression under conditions where the interaction between HIF-1α and newly defined −234/−230 HRE was blocked. In SNU475 cells, which show hypomethylation in the *HK2*-CGI, HK2 expression was observed under normoxic conditions and increased by hypoxic conditions. In contrast, SNU449 cells, which exhibit hypermethylation in that region, did not express HK2, even under hypoxic conditions (Figure 4A). The increase in HK2 expression in SNU475 cells was reversed by si-*HIF1A*-mediated silencing (Figure 4B).

To demonstrate that *HK2*-CGI hypermethylation is a prerequisite for HK2 suppression, we evaluated whether 5-Aza-CdR-induced hypomethylation could reverse HK2 expression. Treatment of SNU475 and SNU449 cells with 5-Aza-CdR caused hypomethylation of the *HK2* promoter (Figure 4C). Because SNU475 cells were originally hypomethylated on the −234/−230 HRE of the *HK2*-CGI, 5-Aza-CdR did not change HK2 expression (Figure 4D left panel). In contrast, SNU449 cells expressed HK2 following treatment with 5-Aza-CdR and hypoxic stimuli (Figure 4D right panel). This observation was the result of increased binding of HIF-1α to the −234/−230 HRE in the *HK2* promoter (Figure 4E). All together, these findings indicate that the *HK2*-CGI hypermethylation suppresses HK2 expression via inhibition of the interaction between HIF-1α and −234/−230 HRE.

### *HK2*-CIMP, a distinct subgroup in HCCs

**Figure 5:**
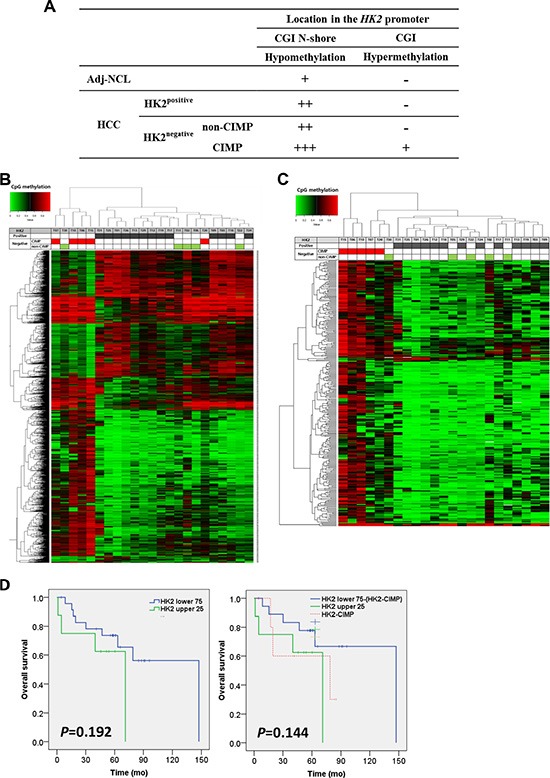
*HK2*-CIMP, a distinct subgroup of HCCs (**A**) The methylation status of *HK2*-CGI and its N-shore, according to HK2 expression. Unsupervised hierarchical clustering of (**B**) the top 1,417 significantly differentially methylated CpG sites between 13 HK2^positive^ and 10 HK2^negative^ HCCs and (**C**) further selected 289 CpG sites located in the promoter CGI which corresponds to TSS1500, TSS200, 5′UTR and 1st Exon. (**D**) Overall survival after surgery was compared using Kaplan-Meier analysis (Left panel) between the HK2 upper 25th percentile (*n* = 8) and HK2 lower 75th percentile (*n* = 24), and (Right panel) between HK2 upper 25th percentile (*n* = 8) and HK2 lower 75th percentile except *HK2*-CIMP HCCs (*n* = 19), which were plotted separately as an *HK2*-CIMP group (*n* = 5).

*HK2*-CIMP HCCs exhibited a different methylation pattern in the *HK2*-CGI (Figure 5A). Because CpG methylation was not altered at a single gene level, as visualized by unsupervised hierarchical clustering analysis (Supplementary Figure S1), we further analyzed CpG sites according to HK2 expression. Most *HK2*-CIMP HCCs exhibited divergent methylation patterns from other HCCs and clustered together (4/5, 80%; Figure 5B). Furthermore, when we sorted and analyzed the CGIs located within the promoter locus by unsupervised hierarchical clustering, we found 289 CpG sites with differential methylation, which enabled us to discriminate *HK2*-CIMP HCCs from other HCCs (Figure 5C).

HK2 expression in glioblastoma multiforme (GBM) tumors was associated with reduced overall survival (OS) of patients [28]. Thus, we examined OS in our set of HCCs according to HK2 expression. Our data did not show a difference in OS between the HK2 upper 25th percentile group (HK2 upper 25; *n* = 8) and the HK2 lower 75th percentile group (HK2 lower 75; *n* = 24) (*P* = 0.192, Figure 5D left panel). We tested whether this result was due the grouping of *HK2*-CIMP HCCs into the HK2 lower 75 group. When *HK2*-CIMP HCCs were plotted as a group in a Kaplan-Meier survival analysis, they displayed a similar OS to that of the HK2 upper 25 group. Moreover, the *P* value was reduced by removing the *HK2*-CIMP HCCs from the HK2 lower 75 group (*P* = 0.144, Figure 5D right panel). These findings suggest that *HK2*-CIMP HCCs should be regarded as a distinct subgroup in HCCs.

## DISCUSSION

**Figure 6:**
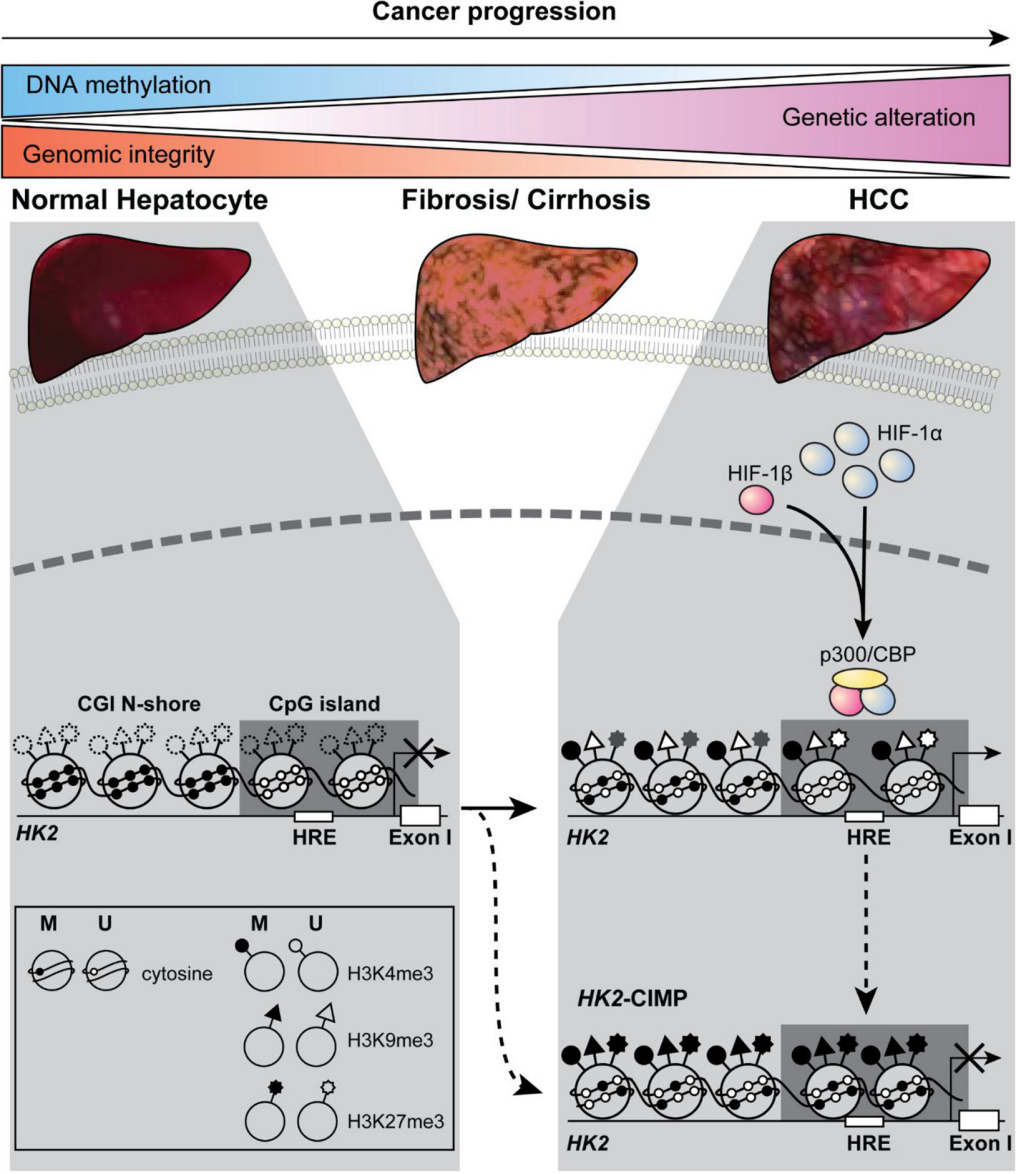
A model for opposing regulatory mechanisms of *HK2* expression, according to the methylation status of the *HK2*-CGI and its N-shore In normal liver cells, the *HK2* promoter is initially hypermethylated (closed circles) at the *HK2*-CGI N-shore and hypomethylated (open circles) at the *HK2*-CGI. In HCCs with HK2 expression, the *HK2*-CGI N-shore is progressively hypomethylated, while the *HK2*-CGI remains unmethylated, as evidenced by active chromatin marks (H3K4me3 high, H3K9me3 low, and H3K27me3 low). These modifications enable HIF-1α to access the HRE in the *HK2*-CGI, resulting in *HK2* expression. In *HK2*-CIMP HCCs, the *HK2*-CGI is methylated and is accompanied by the poised status of chromatin (H3K4me3 high and H3K27me3 high), which inhibits HIF-1α from binding to the HRE in the *HK2*-CGI, resulting in *HK2* suppression. The dashed circles, triangles, and stars denote that the methylation status of H3 lysine was not determined.

HK2 expression offers an advantage to cancer cells by increasing aerobic glycolysis, resulting in an altered metabolic state with an anti-apoptotic effect [2]. Despite this survival benefit, HCCs are heterogeneous in their expression of HK2 [3–5]. Here, we demonstrated that the heterogeneous expression of HK2 was caused by altered CpG DNA methylation at the *HK2* promoter (summarized in Figure 6). In agreement with a recent study of hypomethylation in the promoter CGI N-shore [23], we report, for the first time, the *HK2*-CGI N-shore hypomethylation shows a strong association with HK2 expression in HCCs. Moreover, despite severe *HK2*-CGI N-shore hypomethylation, *HK2*-CGI hypermethylation in *HK2*-CIMP HCCs merely abolished HK2 expression. This observation was the result of a blocked HIF-1α binding site, −234/−230 HRE, a newly defined key regulatory region in the *HK2* promoter. Finally, the correlation of *HK2*- CIMP with clinical outcome suggests that *HK2*- CIMP HCCs could be regarded as a distinct subgroup of HCCs, and *HK2*-CGI hypermethylation, represented by −40 CpG hypermethylation, could be used as a biomarker for predicting prognosis of HCC patients.

The effects of aberrant DNA methylation changes on gene expression have been examined in many cancers, including HCCs [29, 30]. Those studies focused on the differences of mean values of CpG DNA methylation in promoter regions only. Thus, the correlation between methylation changes and gene expression was weaker than expected. However, a recent report on aberrant DNA methylation in HCCs investigated methylation changes according to genomic locations, such as the promoter CGI and its surrounding regions (shore and shelf) [31]. This study revealed that CpG DNA methylation changes were more frequently observed in the promoter CGI shore than in the promoter CGI [31], which may result in altered gene expression [23].

Our observations of global hypomethylation and *HK2*- CGI N-shore hypomethylation are consistent with previous studies [29–31], but the *HK2*-CGI hypermethylation that we observed in *HK2*-CIMP HCCs has not been previously reported. Moreover, to our knowledge, this is the first report of such opposing alterations in the same gene. We observed *HK2*-CGI hypermethylation only in HK2^negative^ *HK2*-CIMP HCCs and in SNU449 cells, which might be explained by a model of *de novo* methylation in cancer [22]. In normal hepatocytes, the *HK2*-CGI remained largely unmethylated, which might be mediated by polycomb repressive complex 2 (PRC2) as observed in other genes [22, 32]. However, in HCCs, unknown factors may cause an increase in EZH2 and G9A expression, resulting in the enrichment of H3K27me3 and H3K9me3. Moreover, the increase in EZH2 or G9A might alter the equilibrium of PRC2 at the *HK2*-CGI, which enables DNMT3s to bind to EZH2 or G9A via SET domain and cause *de novo* methylation. The increased expression of EZH2 and G9A and the enrichment of H3K27me3 and H3K9me3 that we observed in SNU449 cells supports this hypothesis.

When CpG DNA methylation was analyzed according to HK2 expression, most *HK2*-CIMP HCCs were grouped together. Furthermore, when this analysis was restricted to CpGs in the promoter CGI, all *HK2*-CIMP HCCs show a similar methylation pattern. Thus, *HK2*-CIMP HCCs might represent a subgroup having a *de novo* methylation-prone phenotype. Therefore, *HK2*-CGI methylation could be applied as a biomarker for an epigenetic subtyping to suggest potential prognosis of HCC. Our newly found 289 hypermethylated CGIs suggest the molecular mechanism of *de novo* methylation, thus, we are currently investigating this *de novo* methylation using SNU449 cells.

We also defined a cis-acting region −234/−240 HRE and its trans-acting element, HIF-1α. Moreover, 5-Aza-CdR-mediated hypomethylation showed that *HK2*-CGI hypermethylation could directly suppress HK2 expression. However, in some HK2^negative^ non-CIMP HCCs, HK2 was not expressed in spite of the hypomethylation in both *HK2*-CGI and its N-shore. According to our data, the meaning of *HK2*-CGI hypermethylation could be different from that of *HK2*-CGI N-shore hypomethylation. When *HK2*-CGI hypermethylation is present (called *HK2*-CIMP), the *HK2*-CGI hypermethylation is a critical determining factor for HK2 expression. However, when *HK2*-CGI methylation is absent, the regulation of HK2 expression was not only influenced by the methylation status of *HK2*-CGI N-shore, but also influenced by other factors such as the level of HIF-1α expression, and miRNA, which must be further evaluated. To understand the precise regulation of *HK2* expression, we need more understanding of the crosstalk among transcription factors, CpG DNA methylation, and chromatin status. For instance, in normal hepatocytes, HK2 expression was suppressed despite the absence of *HK2*-CGI methylation. This observation may be due to repressed chromatin status or polycomb proteins, which keep CGIs unmethylated by inhibiting *de novo* methylation [22]. Thus, we need to continue to investigate the epigenetic status of the *HK2* promoter and the detailed interactions of related proteins, which could reveal some factors that cause *de novo* methylation in promoter CGIs.

Some limitations to this study should be addressed. First, the correlation of *HK2*-CIMP and HCC progression was not fully evaluated. A recent report showed that HK2-suppressed GBM tumors had more aggressive and metastatic features [28]; however, our results on OS were not significant. These limitations could be overcome through the evaluation with larger sample size of *HK2*-CIMP HCCs. Second, we did not fully address the molecular mechanism of hypomethylation observed in the *HK2*-CGI N-shore, which might share a mechanism with the *HIF1A* promoter hypomethylation [33]. Ten-Eleven Translocation 1 (TET1) regulates the expression of HIF-1α target genes by inducing CpG hypomethylation near an HRE [34]. Interestingly, TET1 interacts with PRC2 [35]. Thus, we could hypothesize that the interactions between TET1, PRC2, and the *HK2*- CGI lead to hypomethylation in the *HK2*-CGI N-shore. Further evaluation and testing of this hypothesis may lead to a generalized mechanism of regulation for other genes that have CGIs in their promoters. Finally, we did not examine the interaction between the *HK2* promoter and Sp1. We observed a decrease of luciferase activity between the −268 and −255 constructs, as reported previously [36], and our ChIP assay data revealed an interaction between Sp1 and this region. Recently, an interaction between Sp1 and HIF- 1α was reported [37, 38]. Thus, further studies may reveal an interaction between Sp1 and HIF-1α in the regulation of HK2 expression.

In conclusion, we identified two oppositional alterations in the *HK2* promoter that regulate *HK2* expression. The first alteration is hypomethylation in the *HK2*-CGI N-shore, which increases *HK2* expression. The second alteration is hypermethylation in the *HK2*-CGI, which contributes to *HK2* suppression via inhibition of the interaction between −234/−230 HRE and HIF-1α. The latter alteration was observed only in *HK2*-CIMP HCC cells. *HK2*-CIMP HCCs represent a subgroup of cells with hypermethylated CGI in the promoter and a poor clinical outcome. Thus, *HK2*-CGI methylation status could serve as a prognostic biomarker.

## MATERIALS AND METHODS

### Patients and tissue samples

Among the patients who underwent a liver resection between 2009 and 2010 at Severance Hospital, Yonsei University College of Medicine, Seoul, Korea, the clinical and histological data were analyzed from 32 HCC patients whose tissue samples were available. Upon authorization from the Institutional Review Board of Severance Hospital (4-2013-0789), snap-frozen tissue samples were provided by the Gene Bank in Severance Hospital.

### Cell culture

Hep3B, SNU449, and SNU475 cells were obtained from the Korean Cell Line Bank (Seoul, Korea) and cultured in RPMI-1640 or Modified Eagle Medium supplemented with 10% FBS (HyClone, Tauranga, New Zealand) at 37°C in a humidified CO_2_ incubator. Hypoxia experiments were conducted in 1% O_2_, 5% CO_2_, and 94% N_2_ overnight. Cells were treated with 5-Aza-2-deoxycytidine (5-Aza-CdR; Sigma-Aldrich, St. Louis, MO, USA) in 6-well plates for 72 h with freshly prepared 5-Aza-CdR.

### HumanMethylation450 BeadChip array

Genomic DNA was extracted from human liver tissues and HCC cell lines (Hep3B, SNU475 and SNU449 cells) using QIAGEN DNA miniprep kits (Valencia, CA, USA) according to the manufacturer's instructions. Then DNA was modified using EZ DNA methylation-direct kits (Zymo Research, Irvine, CA, USA). The HM450 BeadChip array (Illumina, San Diego, CA, USA) analysis was conducted according to the manufacturer's instructions. Methylation levels were reported as β-values, with a range from 0 to 1. All HM450 arrays were processed by LAS Inc. (Daejeon, Korea).

### Bisulfite sequencing

Nested PCRs were performed with bisulfite-treated genomic DNA from Hep3B, SNU475 or SNU449 cells using the primers described in Supplementary Table S1. The resulting amplification pools were cloned into the pCR II vector using a TA cloning kit (Invitrogen Life Technologies, Carlsbad, CA, USA) and sequenced using T7 and SP6 primers.

### Immunoblot analysis

The following antibodies (Abs) were used: anti-HK2 (sc-6521; Santa Cruz, Dallas, TX, USA), anti-DNMT1 (A300-041A; Bethyl Laboratories, Montgomery, TX, USA), anti-DNMT3A (LS-C167495; LifeSpan BioSciences, Seattle, WA, USA), anti-DNMT3B (LS-C128621; LifeSpan BioSciences), anti-G9A (A300-933A; Bethyl Laboratories), anti-EZH2 (LS-C167874; LifeSpan BioSciences), anti-HIF-1α (A300-286A; Bethyl Laboratories), and anti-β-actin (AC-15; Sigma-Aldrich).

### Chromatin immunoprecipitation (ChIP) assay

Cells were fixed with 1% formaldehyde (Sigma-Aldrich) for 20 min. Fixation was quenched with 0.125 M glycine for 10 min. Cells were harvested and lysed, and nuclei were sonicated using a Bioruptor (COSMO Bio, Tokyo, Japan). Chromatin samples were immunoprecipitated with anti-H3 (ab1791; Abcam, Cambridge, UK), anti-H3K4me3 (ab8580; Abcam), anti-H3K9me3 (ab8898; Abcam), anti-H3K27me3 (ab6002; Abcam), anti-HIF-1α, anti-Sp1 (EMD Millipore, Temecula, CA, USA) and control IgG (sc-2027; Santa Cruz) at 4°C overnight, and then proteins were harvested with mouse anti-IgG Ab linked to magnetic protein A&G beads (Dynal, Lake Success, NY, USA). Immune complexes were disrupted, and 2 μl DNA dissolved in 50 μl water was used as template for PCR with the primers described in Supplementary Table S1.

### Promoter luciferase assay

All luciferase reporter constructs were generated by PCR using the described primer sets (Supplementary Table S2) and the pGL3-basic luciferase reporter vector (Promega, Madison, WI, USA). All mutant constructs were generated using a QuikChange Site-Directed Mutagenesis Kit (Stratagene, Santa Clara, CA, USA). Cells were transfected with luciferase reporter constructs including 50 ng pRL-SV40 as a transfection control using Lipofectamine 2000 (Invitrogen Life Technologies). DNA quantities equimolar to 2 μg of the -1921 human *HK2* promoter construct were used for all luciferase reporter constructs. The luciferase activities were measured using the Dual-Luciferase^™^ Reporter Assay System (Promega) and a VICTORTM X4 luminometer (PerkinElmer, Waltham, MA, USA), and were analyzed based on the ratio of Firefly (luciferase constructs): Renilla (pRL-SV40 vector) and normalized to the cell number and transfection efficiency.

### Electrophoretic mobility shift assay (EMSA)

EMSAs were conducted with nuclear extracts from Hep3B cells as described previously [39]. Double stranded −234/−230 HRE or −234/−230 HRE mutant (−234/−230 HREm) probes were labeled with [γ-^32^P] ATP using T4 polynucleotide kinase (New England BioLabs, Ipswich, MA, USA) and purified with a ProberTM column (iNtRON, Gyeonggi-do, Korea). The binding reactions were completed with or without competitors, including −234/−230 HRE or −234/−230 HREm cold probes, and anti-HIF-1α Ab. Protein-DNA complexes were separated from the free probe by electrophoresis on a 5% polyacrylamide gel in 0.25× TBE buffer. After drying, gels were exposed to a Phosphor Imager FLA 7000 (FUJIFILM, Tokyo, Japan) at −70°C overnight.

### Small interfering RNA (siRNA)

The siRNAs targeting *HIF1A* (NM_001530) (Supplementary Table S3, Integrated DNA Technologies, Coralville, IA, USA) were transfected using Lipofectamine RNAiMAX (Invitrogen Life Technologies) according to the manufacturer's protocol.

### Statistical analysis

For statistical analyses, unpaired Student's *t*-tests, Pearson's correlation tests, and Kaplan-Meier analyses were applied as appropriate using SPSS 20 for Windows (IBM, Armonk, NY, USA). All *in vitro* experiments were performed at least three times. Values represent the mean ± SEM. For all experiments, statistical significance is denoted accordingly: *P < 0.05, **P < 0.01, ***P < 0.001.

## SUPPLEMENTARY FIGURES AND TABLES


