# Supplementary material for: Regulation of HK2 expression through alterations in CpG methylation of the HK2 promoter during progression of hepatocellular carcinoma
Source: Oncotarget. 2016 May 30;7(27):41798–810. doi: 10.18632/oncotarget.9723 (PMC5173097; doi:10.18632/oncotarget.9723)
Supplement: Supplementary file 1 [file oncotarget-07-41798-s001.pdf]

Regulation of *HK2* expression through alterations in CpG methylation of the *HK2* promoter during progression of hepatocellular carcinoma

Supplementary Materials

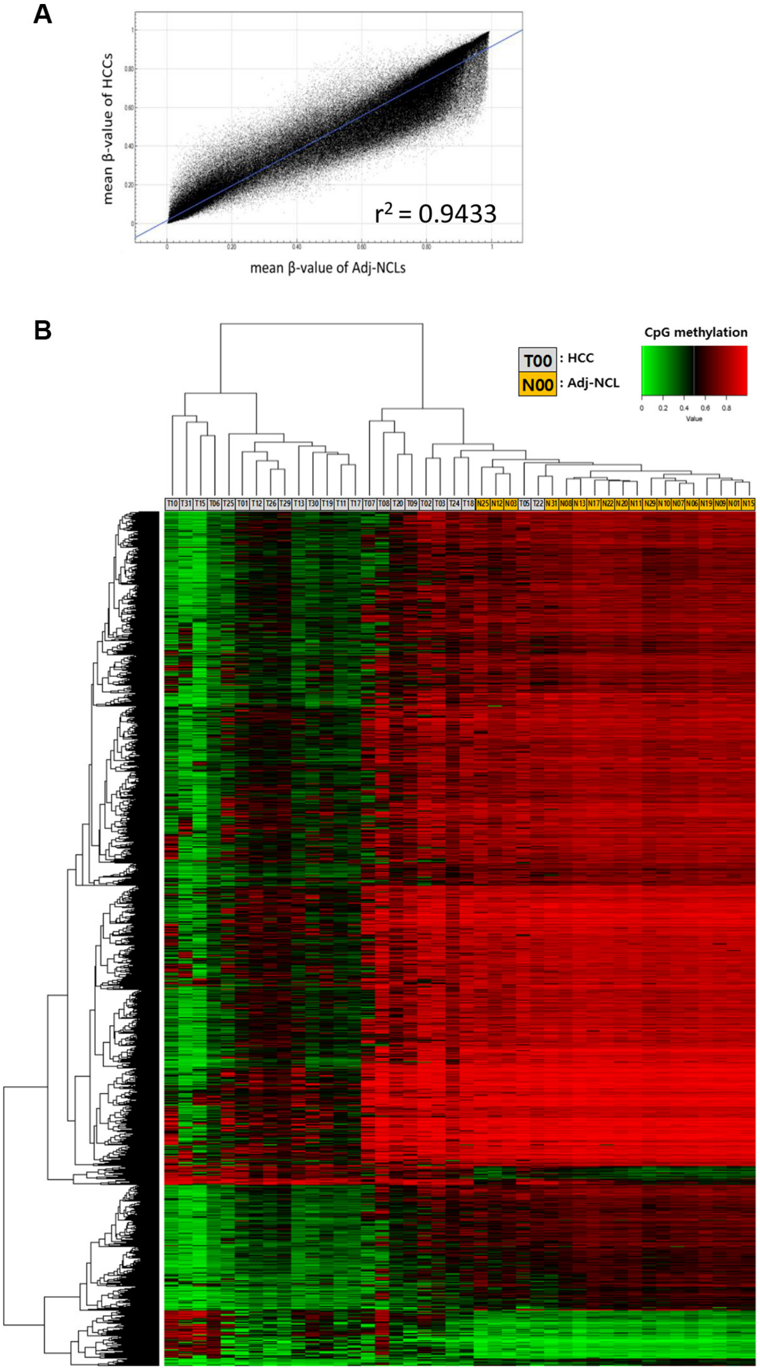

Supplementary Figure S1: Global hypomethylation of CpG DNAs in the HCCs. (**A**) The mean β-values of HCCs and Adj-NCLs were plotted. (**B**) Unsupervised hierarchical clustering of 24 HCCs and 18 Adj-NCLs, by samples and by CpGs showing delta mean β-values between HCC and its Adj-NCLs > ± 0.2 (total 25,330 CpG sites).

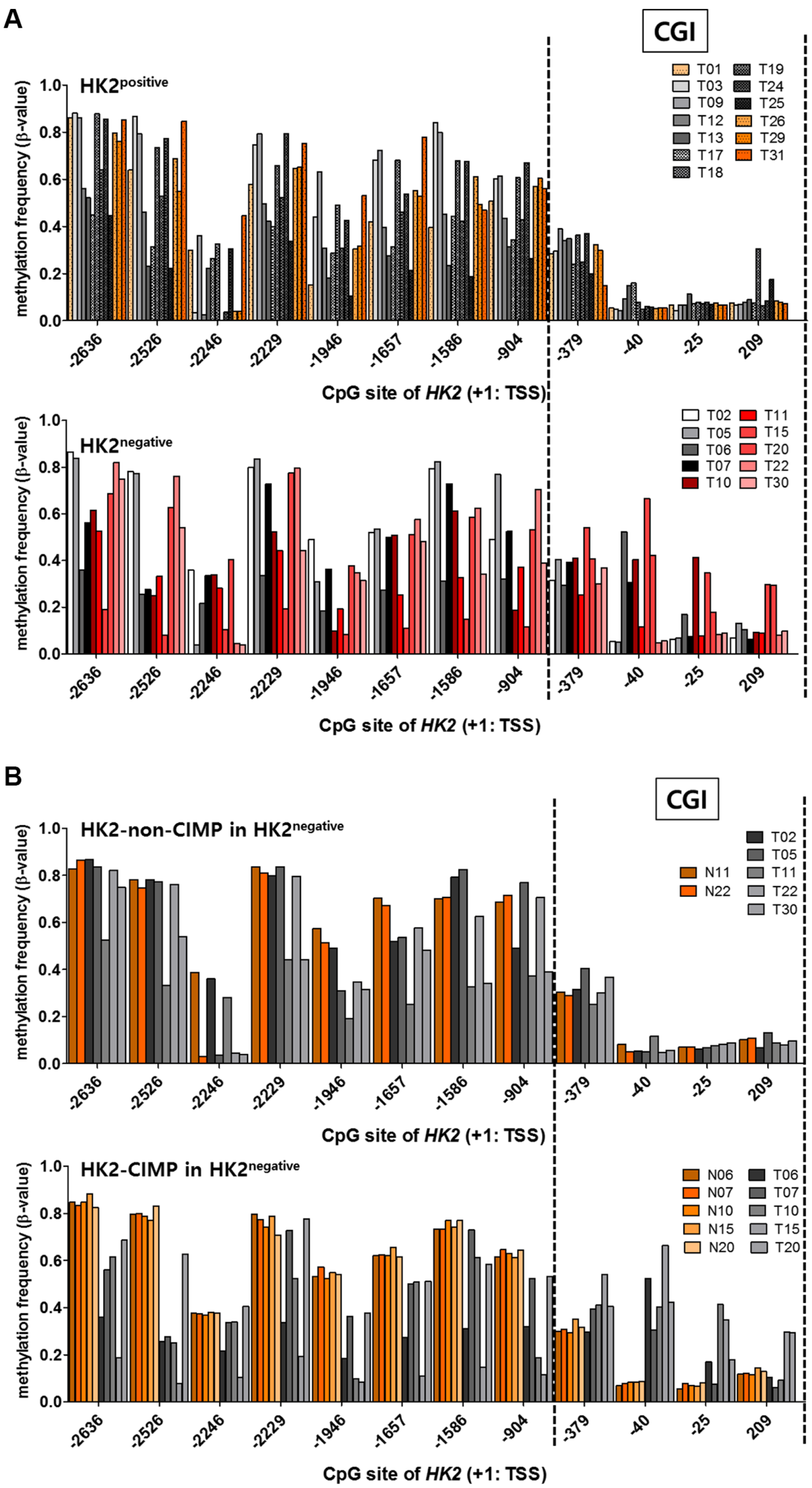

Supplementary Figure S2: The methylation status of the *HK2* promoter according to HK2 expression in HCCs. The methylation status of the *HK2* promoter CpGs was plotted according to HK2 expression: (**A**) HK2^{positive} and HK2^{negative}, (**B**) *HK2*-CIMP HCCs (T06-T20) and *HK2*-non-CIMP HCCs (T02-T30). Their Adj-NCL tissues were co-plotted, respectively.

Supplementary Table S1: Primer sequences

Name of Primer			sequences
**ChIP**	−1135F (CGI-shore)	F	5′-AAGCCTCCTCACCCACCCCA-3′
	−963R (CGI-shore)	R	5′-GGCCCTATTCGCTTGCCCCA-3′
	−302F (CGI)	F	5′-ACCCATAGCCGAGCCTGAC-3′
	−114R (CGI)	R	5′-GCAGCCAATCATCGCTCAC-3′
	+480F (control)	F	5′-TCACGGAGCTCAACCATGAC-3′
	+792R (control)	R	5′-GGAAAAAGGGACTGGGGGAG-3′
**Bisulfite Sequencing[1]**	HK2-BS-F	F	5′-TTATAATAGGTAAGAATTAGGATGTTTTAG-3′
	HK2-BS-1st R	R	5′-TCATACAACAATATAACTTATTAAAAAACT-3′
	HF2-BS-2nd R	R	5′-CCTAAAATAAAACGTATAATCCGTA-3′

[1]Nested PCR was performed due to the low efficiency of PCR

Supplementary Table S2: Primer sequences used to construct luciferase reporter vectors

Name of Primer		sequences
**−965F**	F	5′-GCCTTAAAGTAGAGGGACAAGGG-3′
**−407F**	F	5′-GATCCCGAGATGCCAGACAG-3′
**−337F**	F	5′-GCCCCACACC CCGGGTCC-3′
**−305F**	F	5′-CCCACCCATAGCCGAGCCTGAC-3′
**−268F**	F	5′-TG CGCCTTTCCGTCCCAGCC-3′
**−255F**	F	5′-CCAGCCTT TAGCCACGGACCACA-3′
**−175F**	F	5′-AGGCTGCC GGCTCCGGTGTCTG-3′
**+106R**	R	5′-CTCTCCGATTCACAGGCGCGTGG-3′

Supplementary Table S3: siRNA sequences

Target	No. of siRNA	sequences
HIF1A	1	5′-GGAAGAACUAUGAACAUAAAGUCTG-3′
	2	5′-ACAAUACCCUAUGUAGUUGUGGAAG-3′
	3	5′-CCAGCAGACUCAAAUACAAGAACCT-3′
